# Editorial: The next generation of tools and technologies for studying human neurons in a dish

**DOI:** 10.3389/fncel.2023.1196543

**Published:** 2023-04-14

**Authors:** Thomas M. Durcan, Alison D. Axtman

**Affiliations:** ^1^Early Drug Discovery Unit, Montreal Neurological Institute-Hospital, McGill University, Montreal, QC, Canada; ^2^Structural Genomics Consortium, UNC Eshelman School of Pharmacy, University of North Carolina at Chapel Hill, Chapel Hill, NC, United States

**Keywords:** induced pluripotent stem cells (iPSCs), drug discovery, neuronal model, cilia, dopaminergic neurons, casein kinase 2 (CK2) inhibitors, tau, Alzheimer's disease

## Introduction

Despite their prevalence, disorders of the brain, including neurodevelopmental, neurodegenerative, and other mental illnesses, have historically been some of the most challenging to treat. The enormous economic burden coupled with a paucity of curative therapies highlights an urgent need for new approaches and targets to tackle these diseases. One biologically relevant and transformative method that has been employed is the use of induced pluripotent stem cells (iPSCs) to generate neurons that closely mimic those found within the human brain (Mohamed et al., 2019). Advances in this area have enabled investigators to readily grow many of the cell types found within the human brain on a dish, increasing our understanding of mechanisms and targets that are implicated in these diseases and helping to facilitate translational efforts (Fermini et al., 2018).

With this in mind, this Research Topic is aimed at capturing new approaches and ideas for studying neurons in a dish. The goal is to broaden current understanding related to iPSCs and other models and processes that can be applied to developing and studying human neurons in a dish. Chemical tools, including heterobifunctional, chemical probes, and small molecules are another essential piece of this emerging field as they represent pre-clinical leads for new therapeutics. The issue currently includes six papers, which are a mixture of original research articles and reviews focused on iPSC-derived neuronal models and the characterization of new therapies for Alzheimer's disease (AD). The papers are from several fields across reproducibility, cilia, oxidative stress, E3 ligases, protein degraders, and neuroinflammation. All contributions to this Research Topic focus on one or more of the research areas highlighted above, as discussed further in the sections that follow.

## Improvement of iPSC-derived neuronal models

As a first step in better understanding the brain and gaining mechanistic insight into why brain disorders arise, access to human brain cells and tissues is essential. In their review, Knock and Julien address this notion, defining how previous and current work with animal and primary patient tissue models can better inform our applications of patient-derived iPSC models for discovery and translational work on the brain. As part of their discussions, they outline culture models of human neural tissue, while defining advantages over animal models and key challenges that remain to be overcome. By highlighting studies that combine animal models and human neural cell culture techniques, they strive to lay out how the use of these orthogonal model systems can produce more reproducible, physiological, and clinically relevant data than either approach alone, concluding with advancement on iPSC-derived neurons that can potentially one way be directed into clinical cellular replacement programs.

## Developmental cues and factors at the core of developing physiological models of the brain

While the review from Knock and Julien highlighted many of the key aspects around building these models of the brain with stem cells, two other reviews focus on the impact of developmental signaling in developing a specific neuronal subtype (as discussed by Ni and Ernst), or the influence of primary cilia as a central site through which many of the neurodevelopmental pathways proceed (as discussed by Rocha and Prinos) and how they influence the formation of different neuronal subtypes and brain regions. Both reviews raise important points around neurodevelopmental signaling and highlight how to better build models of the brain from patient-derived iPSCs. We need to first understand the developmental pathways that are involved in the formation of the human brain itself. By doing so, researchers can now move toward deriving dopaminergic neurons that more physiologically resemble the dopaminergic neurons of the substantia nigra that are progressively lost in Parkinson's disease (Ni and Ernst). In studying neurodevelopmental disorders and better understanding how neurons form from iPSCs, cilia are at the forefront of these processes, acting as mediators of WNT, Sonic Hedgehog (SHH), and PDGF, all pathways that are critical for neurogenesis signaling (Rocha and Prinos). Taken together, iPSCs provide an entry point to making any cell type found in the human brain, and through a better understanding of the developmental cues and using previous human and animal models, it can guide us toward better understanding and treating disorders of the brain. Taking this into account, the other reviews discuss how these models can be applied in the development of new therapies, with a focus on Alzheimer's disease.

## Characterization of new therapies for Alzheimer's disease

This section contains three papers aimed at developing new therapeutic strategies to combat Alzheimer's Disease (AD) that rely, in part, on a combination of small molecules and neuron-based models (Potjewyd and Axtman; Silva et al.; Mishra et al.). Included are two original research articles (Silva et al.; Mishra et al.) and one review (Potjewyd and Axtman), which highlight how small molecules and/or bifunctional molecules are gaining traction as unconventional and promising pre-clinical approaches that target AD-propagating pathways.

Harnessing the impact of kinase inhibitors, Mishra et al. used a casein kinase 2 (CK2) chemical probe called SGC-CK2-1 to explore the role of CK2 in modulating neuroinflammation. Combining this potent and selective CK2 inhibitor with iPSC-derived microglia-like cells (MGLs) enabled the study of this pleiotropic kinase (CK2) in the innate immune cells of the central nervous system. The impact of SGC-CK2-1 and a CK2-targeting clinical candidate, CX-4945, were compared in wild-type and presenilin-1 (PSEN1) mutant MGLs, and while both suppressed proinflammatory cytokine expression, SGC-CK2-1 was much more effective than CX-4945 and at much lower doses. This study underscores the power of using potent and selective chemical probes to match a disease-relevant phenotype to its protein target as well as the disease-altering impact that kinase inhibitors could have on disorders of the brain.

E3 ligases are another area of recent and robust interest. Silva et al. employed patient-derived iPSCs to derive a neuronal model of tauopathies, which include AD and frontotemporal dementia (FTD), characterized by an accrual of aberrant forms of the protein tau in the brain. An FTD-patient iPSC-derived neuron expressing a tau variant or mutation was employed to drive medicinal chemistry aimed at developing a tau-targeting degrader. A lead compound (QC-01-175) was identified and degradation of mutant tau by three advanced compounds was demonstrated, which validated the screening approach and set the stage for future efforts. As a divergence from proteins like tau that are well-studied and hallmark proteins of AD, Potjewyd and Axtman surveyed less-studied proteins implicated in AD pathology. With a similar endpoint of harnessing the power of E3 ligases for bifunctional molecule development in mind, this review highlighted several E3 ligases that have differential expression in AD brains when compared to those from normal, non-demented controls. Chemical modulators of these AD-implicated E3 ligases were presented as a method via which to modulate their function as well as specific examples of the use of cultured neurons to delineate E3 ligase biology. Collectively, these articles highlight the promise of using bifunctional molecules (such as PROTACs) as a novel therapeutic modality to slow or halt AD pathology.

## Perspectives

In conclusion, this Research Topic provided a suite of six reviews discussing how iPSCs can be applied when compared to earlier models, developmental cues, and signaling that can inform our approach in generating neuronal models on a dish. By generating these models, the topic concludes with a focus on distinct targets and small molecules that can be combined with these iPSC-derived models of the brain toward better understanding and treating disorders of the brain.

## Author contributions

All authors listed have made a substantial, direct, and intellectual contribution to the work and approved it for publication.
